# Machine learning models for the prediction of levodopa response to tremor in Parkinson’s disease

**DOI:** 10.3389/fnagi.2025.1690155

**Published:** 2026-01-05

**Authors:** Fangfei Li, Shinuan Lin, Rui Yan, Yusha Cui, Kang Ren, Zhonglue Chen, Lingyan Ma, Tao Feng

**Affiliations:** 1 Center for Movement Disorders, Department of Neurology, Beijing Tiantan Hospital, Capital Medical University, Beijing, China; 2 Department of Neurology, Beijing Chaoyang Hospital, Capital Medical University, Beijing, China; 3 GYENNO Science Co., Ltd., Shenzhen, China; 4 HUST-GYENNO CNS Intelligent Digital Medicine Technology Center, School of Artificial Intelligence and Automation, Huazhong University of Science and Technology, Wuhan, China; 5 China National Clinical Research Center for Neurological Diseases, Beijing, China

**Keywords:** Parkinson’s disease, machine learning, levodopa responsiveness, tremor, support vector machine, validation

## Abstract

**Objectives:**

To develop and validate machine learning models to predict levodopa responsiveness of tremor in Parkinson’s disease (PD) patients.

**Methods:**

A total of 197 PD tremor patients underwent Levodopa Challenge Tests and were classified as having levodopa-responsive or levodopa-resistant tremor. Clinical and electromyogram (EMG) tremor analysis variables were recorded. The dataset was randomly divided into a training set (80%) and a test set (20%). To distinguish between the two groups, Support vector machine (SVM), random forest (RF), and logistic regression (LR) models were developed using training data. The optimal model was validated on test data. Calibration and decision curve analyses assessed model reliability and clinical utility.

**Results:**

Among 197 patients, 95 had levodopa-responsive tremor, and 102 had levodopa-resistant tremor. The SVM model showed the best performance, achieving an accuracy of 81.5% in five-fold cross-validation, with a Kappa score of 0.624, sensitivity of 84.3%, specificity of 77.9%, and an area under the curve (AUC) of 0.850. Performance remained consistent on test data, with 82.5% accuracy, 0.653 Kappa, 93.8% sensitivity, 75% specificity, and 0.896 AUC. The best model incorporated 6 predictors: resting tremor score, rigidity/tremor ratio, postural and kinetic tremor score, disease duration, the Movement Disorder Society’s Unified Parkinson’s Disease Rating Scale III (MDS-UPDRS III) /disease duration, and supine diastolic blood pressure (DBP).

**Conclusion:**

The SVM model, incorporating six key indicators, holds significant potential for predicting levodopa responsiveness in PD tremor, offering a valuable tool for the precise treatment of tremor in PD patients.

## 1 Introduction

Parkinson’s disease (PD) is characterized by dopamine depletion, resulting in symptoms such as akinesia, rigidity, and tremor. Tremor, which affects approximately 75% of PD patients, is one of the most important motor symptoms of the disease (Helmich et al., 2012). Levodopa remains the most effective treatment for PD, but its effect on tremor varies widely among patients. Compared with akinesia and rigidity, tremor tends to respond less effectively to dopaminergic therapy (Fishman, 2008). While some patients achieve near-complete tremor suppression, approximately 20–30% exhibit dopamine-resistant tremor even with optimized high-dose therapy (Solida et al., 2002). This therapeutic unpredictability leads to prolonged “trial-and-error” dosing, delaying effective symptom control by months or years. The reasons for this variability in tremor response to levodopa remain unclear. Given the significant interindividual variability in tremor response to levodopa, there is an urgent need to identify biomarkers that can predict levodopa efficacy and allow for personalized PD tremor treatment. Traditionally, the levodopa challenge test (LCT) has been used to assess the efficacy of levodopa. However, significant inconsistencies exist in its implementation and interpretation across different medical centers (Pieterman et al., 2018). LCT requires a lengthy evaluation period and can cause side effects such as dizziness, nausea, vomiting, and hypotension. Consequently, this methodology presents notable challenges, including patient discomfort, substantial time investment, and significant resource allocation for clinical facilities, while also posing non-negligible procedural risks. These limitations underscore the critical need for developing efficient predictive models, including valid predictors to evaluate levodopa responsiveness to tremor that mitigate these inherent drawbacks while maintaining diagnostic accuracy.

While numerous clinical factors have been identified as being associated with levodopa responsiveness, the factors influencing the responsiveness of PD tremor to levodopa remain inconsistent in the literature. A previous study showed that patients with dopamine-responsive tremor tended to have more severe disease, longer disease duration, and a higher incidence of dyskinesia (Zach et al., 2020). In another study, a comparison between dopamine-resistant and dopamine-responsive groups revealed statistical differences in gender and levodopa equivalent daily dose (LEDD), but no significant differences in disease duration, Hoehn and Yahr scale (H-Y stage), or Mini-Mental State Examination (MMSE) scores (Dirkx et al., 2019). A separate study focused on predicting clinical factors for dopaminergic treatment response in PD patients with resting tremor. It found that age was significantly different between the responsive and non-responsive groups, while no differences were observed in gender, disease duration, or levodopa dosage. The dopamine-responsive group had significantly higher scores for tremor, rigidity, akinesia, and postural instability, as well as a higher total Movement Disorder Society’s Unified Parkinson’s Disease Rating Scale III (MDS-UPDRS III) score and H-Y stage, compared to the non-responsive group (Sung et al., 2008). More critically, these studies universally neglect autonomic biomarkers—despite some of the PD patients exhibiting orthostatic hypotension and established correlations between sympathetic denervation and tremor-dominant subtypes. Previous findings demonstrate that supine diastolic blood pressure (DBP) reflects central autonomic regulation, potentially modulating tremor circuits through noradrenergic pathways, providing a novel physiological rationale for feature selection. In addition to clinical factors, electromyogram (EMG) tremor analysis has been widely used to measure the difference in dominant frequency and rhythm pattern of tremor between different types of PD tremor. Previous studies have shown that kinetic tremor, which tends to have a higher frequency, is less responsive to dopaminergic therapy than classic resting tremor (Dirkx et al., 2018; Pirker et al., 2023). However, features of EMG tremor analysis have not been investigated as predictors before. Because there are currently inconsistent univariate and multivariate results, further screening and confirmation of predictors is required. New predictive models are therefore urgently needed.

In contrast to traditional predictive models, machine learning approaches consider interregional correlations, which improves the accuracy of the model. Moreover, newer machine learning algorithms have outperformed traditional linear models, such as logistic regression or Cox regression, in handling non-linear and high-dimensional data. This highlights the importance of applying innovative machine learning methods that can effectively capture non-linearity and complex data in clinical settings. Currently, there are no machine learning predictive models for the responsiveness of PD tremor to levodopa.

In this study, we developed machine learning models using both clinical data and features extracted from EMG analysis to predict the levodopa response in PD tremor. Furthermore, in order to verify the validity of the model, independent test data were employed in the study to validate the final model.

## 2 Materials and methods

### 2.1 Subjects

A total of 197 patients diagnosed with idiopathic PD were enrolled from the Movement Disorder Centre, Department of Neurology, Beijing Tiantan Hospital. Participants were included based on the following criteria: (1) The diagnosis of PD was based on the 2015 MDS diagnosis criteria (Postuma et al., 2015); (2) at least one of the following MDS-UPDRS III items had a score of ≥1: Postural tremor of limbs, Kinetic tremor of limbs, resting tremor amplitude, and Constancy of resting tremor. The exclusion criteria were as follows: (1) a history of any brain surgery or neuromodulation therapy (i.e., DBS); (2) extreme physical disability that impairs mobility assessment; and (3) impaired cognitive function or mental problems leading to a failure to complete all the procedures. Written informed consent was obtained from all participants. This study was approved by the Institutional Review Board of Beijing Tiantan Hospital, Capital Medical University (KY2024-037-02).

### 2.2 Data collection

A comprehensive set of demographic data, clinical data, motor symptoms, non-motor symptoms and EMG tremor analysis was assessed under the guidance of movement disorder specialists. Only complete cases (i.e., data without missing values) were included in the analysis.

#### 2.2.1 Demographic and clinical features

Sex, age, onset age, disease duration, and medication history were recorded by inquiring about the case history. Disease staging was assessed by the H-Y stage.

#### 2.2.2 Motor symptoms features

Objective motor symptoms were assessed using the MDS-UPDRS III. In order to reflect different aspects of MDS-UPDRS III in OFF state, the following sub-symptoms and sub-scores were calculated: tremor score (sum of scores on items 3.15–3.18), rigidity score (sum of scores on item 3.3), postural instability score (sum of scores on items 3.9–3.13), hypokinesia score (sum of scores on items 3.4–3.8 and 3.14), midline function score (sum of scores on items 3.1, 3.2, 3.9–3.14), resting tremor score (sum of scores on items 3.17 and 3.18), postural and kinetic tremors score (sum of scores on items 3.15 and 3.16), bradykinesia upper extremity score on more affected side (MAS) [sum of scores on items 3.4 (MAS), 3.5 (MAS), and 3.6 (MAS)], and lower limb bradykinesia score (sum of scores on items 3.7 and 3.8). These definitions are summarized in Table 1 (Goetz et al., 2008). The MAS was determined based on the sum of item scores of the MDS-UPDRS III (3.15; 3.16; 3.17 RUE, LUE, RLE, LLE). If the sum of the above item scores was the same for the left and right side, then the MAS was determined based on the sum of the scores of all the left sided and right sided item on MDS-UPDRS III (3.3 RUE, LUE, RLE, LLE; 3.4; 3.5; 3.6; 3.7; 3.8; 3.15; 3.16; 3.17 RUE, LUE, RLE, LLE). In order to reflect disease progress, several features, including MDS-UPDRS III/disease duration, postural instability/disease duration, hypokinesia/disease duration, tremor /disease duration, and rigidity /disease duration (Table 1), were calculated.

**Table 1:** Patient characteristics.

Time	Feature types	Feature	Explanations
Before LCT	Measured features	Age	Age
		Onset age	Age at disease onset
		Sex	Sex
		Disease duration	Disease duration
		Standing DBP	Diastolic blood pressure in standing
		Standing SBP	Systolic blood pressure in standing
		Supine DBP	Diastolic blood pressure in supine
		Supine SBP	Systolic blood pressure in supine
		Drug type	0: Levodopa and Benserazide Hydrochloride Tablets; 1: Levodopa and Benserazide Hydrochloride Tablets and Carbidopa and Levodopa Sustained-release Tablets; 2: Carbidopa and Levodopa Sustained-release Tablets
		HY	Hoehn-Yahr stage; Disease staging
		LED	levodopa equivalent dose
	Calculated features	MMSE	MMSE total score
		MDS-UPDRS III (OFF)	MDS-UPDRS III total score before LCT
		ΔSBP	Supine SBP—Standing SBP
		ΔDBP	Supine DBP—Standing DBP
		OH	ΔDBP> = 10|ΔSBP> = 20 ~ OH = 1, has orthostatic hypotension; ΔDBP<10 &ΔSBP<20 ~ OH = 0, does not have orthostatic hypotension
		Midline score	sum of scores on items 3.1, 3.2, 3.9–3.14 of MDS-UPDRS III
		Resting tremor score	sum of scores on items 3.17 and 3.18 of MDS-UPDRS III
		Rigidity score	sum of scores on item 3.3 of MDS-UPDRS III
		Bradykinesia upper extremity score (MAS)	sum of scores on items 3.4 (MAS), 3.5 (MAS), and 3.6 (MAS) of MDS-UPDRS III
		Postural and kinetic tremors score	sum of scores on items 3.15 and 3.16 of MDS-UPDRS III
		Lower limb bradykinesia score	sum of scores on items 3.7 and 3.8 of MDS-UPDRS III
		Postural instability score	sum of scores on items 3.9–3.13 of MDS-UPDRS III
		Hypokinesia score	sum of scores on items 3.4–3.8 and 3.14 of MDS-UPDRS III
		Tremor score (OFF)	sum of scores on items 3.15–3.18 of MDS-UPDRS III
		Rigidity/tremor ratio	Rigidity score/tremor score (OFF)
		MDS-UPDRS III/disease duration	MDS-UPDRS III (OFF)/Disease duration
		Postural instability/disease duration	postural instability score/Disease duration
		Hypokinesia/disease duration	Hypokinesia score/Disease duration
		Tremor/disease duration	tremor score (OFF)/Disease duration
		Rigidity/disease duration	Rigidity score/Disease duration
After LCT	Calculated features	MDS-UPDRS III (ON)	MDS-UPDRS III total score at peak period after LCT
		Tremor score (ON)	tremor score at peak period after LCT
		ΔLR MDS-UPDRS III	MDS-UPDRS III (OFF)-MDS-UPDRS III (ON)
		%LR MDS-UPDRS III	ΔLR MDS-UPDRS III/MDS-UPDRS III (OFF)
		ΔLR tremor score	tremor score (OFF)-tremor score (ON)
		%LR tremor score	ΔLR tremor score/tremor score (OFF)

H-Y stage, Hoehn-Yahr stage; LED, levodopa equivalent dose; DBP, diastolic blood pressure; SBP, systolic blood pressure; OH, orthostatic hypotension; MMSE, Mini-Mental State Examination; MDS-UPDRS III, the Movement Disorder Society’s Unified Parkinson’s disease Rating Scale III; MAS, most affected side.

#### 2.2.3 Non-motor symptom features

We measured blood pressure (BP), including supine and standing systolic blood pressure (SBP) and diastolic blood pressure (DBP). Orthostatic hypotension (OH) was also recorded (Freeman et al., 2011). MMSE was used to assess global cognitive impairment.

#### 2.2.4 EMG tremor analysis features

A six-channel Nicolet EDX system (Nicolet company, US) with 4 pairs of EMG surface electrodes and 2 accelerometers was used in this study. During the analysis of the upper limbs, the data were recorded in four states: (1) at rest: patients sat in an armchair with their forearms resting on the armrests, leaving the hands free in the air to measure hand tremors; (2) in a posture: patients raised their arms in front of their body with their wrists parallel with the ground; (3) in a kinetic: patients raised their arms in front of their body with their finger pointing to the nose; and (4) holding 1,000 g: patients held their arms in front of them while holding 1,000 g sandbags. Each state was recorded for 30 s. During the analysis of the lower limbs, the data were recorded under two states: (1) at rest: patients sat calmly in a chair with their feet flat on the ground and relaxed completely; and (2) in a posture: patients sat in a chair with their toes touching the ground and heels suspended. Each state was recorded for 30 s. The dominant frequency and rhythm pattern of tremor were measured under four conditions: at rest, posture, kinetic, and holding 1 kg conditions. These features were constructed as categorical variables to represent clinically relevant distinctions in tremor characteristics based on EMG data, as summarized in Table 2. Tremor frequency features were quantitatively obtained from the acquisition system, which automatically computed the dominant frequency of EMG signals. The continuous frequency values were then discretized into clinically meaningful ranges following established criteria from Helmich’s research (Helmich et al., 2012). 0: Not seen—No resting tremor was detected (clinically observed by a movement disorder specialist). 1: Irregular jerks—Inconsistent or sporadic movements, not classifiable as rhythmic tremor (clinically observed by a movement disorder specialist). 2: <4 Hz—Slow-frequency for resting tremor. 3: [4–6] Hz—Typical frequency range for resting tremor. 4: >6 Hz—Higher frequency for resting tremor. Thus, the raw frequency values were directly computed by the instrument, while the categorical thresholds (<4 Hz, 4–6 Hz, >6 Hz) were defined according to published clinical literature to facilitate consistent interpretation and modeling. Rhythm pattern features were determined through a movement disorder specialist’s interpretation of EMG recordings. Both the agonist and antagonist muscles were analyzed simultaneously to assess their activation relationship. This feature reflects the rhythmic relationship between muscle groups during tremor episodes and was encoded into five clinically relevant categories, as shown below: 0: Not seen—No tremor or rhythmic activity detected. 1: Irregular jerks—Inconsistent, arrhythmic muscle activations not forming a clear pattern. 2: Alternation—Alternating contractions between muscle groups (e.g., agonist–antagonist), often characteristic of Parkinsonian tremor. 3: Synchronization—Simultaneous activations across muscle groups, indicating a different tremor mechanism. 4: Alternation + Synchronization—A mixed pattern, where both alternating and synchronous bursts are observed within the same tremor episode. This combined approach ensures that the EMG-derived features incorporate both objective signal metrics and clinically validated classifications, supporting reproducibility and interpretability in subsequent analyses.

**Table 2:** EMG features.

Feature types	Explanation
Resting tremor frequency (HZ)	
0	Not seen
1	Irregular jerks
2	<4
3	[4, 6]
4	>6
Posture tremor frequency (HZ)	
0	Not seen
1	Irregular jerks
2	<4
3	[4, 9]
4	>9
Kinetic tremor frequency (HZ)	
0	Not seen
1	Irregular jerks
2	<4
3	[4, 9]
4	>9
Holding condition tremor frequency (HZ)	
0	Not seen
1	Irregular jerks
2	<4
3	[4, 9]
4	>9
Rhythm pattern	
0	Not seen
1	Irregular jerks
2	Alternation
3	Synchronization
4	Alternation + Synchronization

### 2.3 Outcome

All participants underwent a levodopa challenge test (LCT) in accordance with published guidelines (Albanese et al., 2001; Feng et al., 2009). The test was conducted in the morning, with withdrawal from anti-Parkinsonian drugs, to ensure a sufficient washout period. The dose for LCT was set gradually until a dose equivalent to 150% of the usual morning levodopa equivalent dose (LED). The drug type and LED were recorded. The MDS-UPDRS III was administered initially in the OFF condition and then repeated after administration. The analysis focused on the highest levodopa responsiveness recorded for each patient. ON and OFF tremor scores were evaluated for each participant, and levodopa tremor responsiveness was calculated as the difference ([OFF–ON]; ΔLR tremor score) and percentage change ([OFF–ON]/OFF*100%; %LR tremor score).

Cluster analysis was performed to establish a classification criterion that categorized PD patients into either levodopa-responsive tremor or levodopa-resistant tremor groups (see Online Resource). Based on the results of the cluster analysis, the following criterion was defined: if a participant had a ΔLR tremor score greater than 3, they were classified as having levodopa-responsive tremor; otherwise, they were classified as having levodopa-resistant tremor.

### 2.4 Model deployment and validation

#### 2.4.1 Training and independent test split

The dataset was randomly divided into a training set (80%) and an independent test set (20%). The proportion of patients with levodopa-responsive tremor did not differ significantly between the training and test sets. All features were derived from assessments conducted in the OFF-medication state, including all ‘Before LCT’ features listed in Table 1 and EMG features collected prior to levodopa administration. Feature analysis and selection were conducted using the training data. Five-fold cross-validation was employed to tune hyperparameters and assess model performance within the training set. The independent test set was then used to evaluate the final model developed from the training data.

#### 2.4.2 Model building and cross-validation

Two steps were performed for feature selection before model building. Step 1: Pairwise Pearson correlation of the features was calculated to remove features with high correlation (threshold 0.6) with another (only one feature was kept for each pair that has a high correlation with each other), following the High-correlation Features Removal Rule mentioned in the previous study (Lin et al., 2023). Step 2: Feature permutation importance was determined using the RF technique (Breiman, 2001), ranking the features retained from Step 1 based on their importance scores, measured by the mean decrease in Gini (MD Gini). A higher MD Gini value indicates greater feature importance, and features were ranked from highest to lowest accordingly.

All classifiers were evaluated using the same ordered feature list derived from the RF permutation-importance ranking, ensuring comparability across models. Classification models were developed using Random Forest (RF), support vector machine (SVM), and logistic regression (LR) techniques to predict whether a patient would be classified as levodopa-responsive tremor or levodopa-resistant tremor. The models were evaluated using the top K features, starting with the top five features (*K* = 5). The decision to begin with five features, rather than fewer, was made to prevent potential underperformance associated with using only a very limited set of features (e.g., 1, 2, 3, or 4 features), which may not sufficiently capture the complexity of the dataset. Additional features were then incrementally added one at a time until all features retained from Step 2 were included in the model, to determine the optimal set of features for the best predictive model performance (Liu et al., 2024; Zhu et al., 2024). At each feature configuration (each value of K), hyperparameter tuning was conducted via grid search within the training set during cross-validation. For the SVM with a Radial Basis Function (RBF) kernel, the hyperparameter grid included penalty parameters (C) ranging from 0.2 to 0.6 in steps of 0.05, and sigma values ranging from 0.02 to 0.06 in steps of 0.01. These ranges were selected to balance model flexibility and generalization capacity, ensuring a comprehensive exploration of decision boundary smoothness and margin settings. For the RF model, the parameter grid consisted of ntree values of 200 and 250, node_size values of 2, 3, 4, and 5 (minimum terminal node size), and mtry values corresponding to *floor* ( $\sqrt{p}$ ) and *ceiling* ( $\sqrt{p}$ ), where *p* denotes the total number of features considered. These ranges were chosen to explore the balance between model complexity and overfitting risk while maintaining computational efficiency. Each algorithm underwent independent feature selection and hyperparameter tuning.

Performance metrics—accuracy, Cohen’s Kappa, sensitivity, specificity, positive predictive value (PPV), negative predictive value (NPV), and receiver operating characteristic area under the curve (AUC_ROC) were calculated for each fold and averaged across five folds. The combination of feature subset and hyperparameters that achieved the highest performance during cross-validation was selected as the optimal configuration for that algorithm. Model performances were then compared across algorithms within cross-validation. The feature, hyperparameter, and model techniques combination corresponding to the model with the highest five-fold cross-validation metrics was selected. This optimal model was then trained on the entire training set using the chosen feature and hyperparameter combination. In our study, we set levodopa-responsive tremor as the positive case, while levodopa-resistant tremor was the negative case. The threshold probability $p_{t}$ was set as 0.5, which was a cut-off value to define when a patient is positive (the patient was regarded as positive when the model predicted probability for that patient was ≥ $p_{t}$ ).

#### 2.4.3 Independent test data validation

The independent test set was applied to evaluate the final model that was built on the entire training data. The model performance evaluation metrics mentioned above were also applied to check the predictive model performance with the independent test data.

#### 2.4.4 Calibration plot and decision curve analysis

Calibration plots and decision curve analysis (DCA) were conducted on the best-performing model using both the training and test datasets to evaluate its clinical utility and predictive reliability. Calibration refers to how well the predicted outcomes align with the observed values (Van Calster et al., 2019). Good calibration indicates that the model accurately estimates risks without overestimating or underestimating them. DCA aimed to address the limitations of traditional statistical metrics (e.g., discrimination and calibration) by trying to answer whether the model should be used in clinical practice.

#### 2.4.5 Additional stability and variability analysis

To further assess the robustness and variability of model performance, an additional repeated 5-fold cross-validation procedure (20 repetitions; 100 resamples) was performed. This analysis was conducted only for performance stability evaluation and did not contribute to model selection, feature selection, or parameter tuning, which were performed exclusively within the primary 5-fold cross-validation on the training set. For each resample, Accuracy, Kappa, Sensitivity, Specificity, PPV, NPV, and AUC were computed. The distributions of these metrics were compared across SVM, RF, and LR using paired t-tests and Wilcoxon signed-rank tests to examine whether differences in mean accuracy were statistically significant.

### 2.5 Statistical analysis

Statistical analyses were conducted using R software version 4.0.2. The statistical significance level was set as 0.05 for all tests. To assess the adequacy of the sample size, a stratified power analysis was performed. The calculation was based on a two-sample *t*-test with the actual group sizes (95 levodopa-responsive vs. 102 levodopa-resistant tremor), a moderate effect size (Cohen’s *d* ≈ 0.5), a significance level of *α* = 0.05, and a two-sided test. The analysis yielded a statistical power of over 80%, confirming that the sample size was sufficient to detect a moderate between-group difference. Statistical tests were selected according to the data type and distribution of each variable. The Shapiro–Wilk test was performed to check if a continuous variable followed a normal distribution. A continuous variable was presented as mean (standard deviation) if it was normally distributed, and an unpaired two-tailed t test was employed for comparison between the levodopa-responsive tremor and levodopa-resistant tremor groups. If a continuous variable was not normally distributed, it was presented as the median [Q1, Q3], where Q1 and Q3 correspond with the 25th percentile and 75th percentile, respectively, and the Wilcoxon rank sum test was employed. Chi-square test was employed to compare categorical variables between levodopa-responsive tremor and levodopa-resistant tremor groups, and categorical variable was presented as numbers (percentages).

Univariate odds ratios (ORs) and their corresponding 95% confidence intervals (CIs) were used to quantify the relationship between our tremor levodopa response outcome associated with each variable. The ORs represented the change in the odds of levodopa-responsive tremor for each one-unit increase in the variable for continuous variables, while they reflected the change in odds of levodopa-responsive tremor relative to the selected reference group for categorical variables.

## 3 Results

### 3.1 Participants characteristics

A total of 197 participants were included in this study. The main demographic and clinical characteristics of the participants are presented in Table 3. There was no statistically significant difference in the main demographic and clinical characteristics between the training data (157 participants) and the test data (40 participants). In the training data, 79 participants were classified as having levodopa-responsive tremor, while 78 participants were classified as levodopa-resistant tremor.

**Table 3:** Main characteristics of all the participants.

Feature	Overall	Train	Test	*p* ^*^
	197	157	40	
Levodopa-responsive tremor, *n* (%)	95 (48.20)	79 (50.30)	16 (40.00)	0.323
Sex: Female, *n* (%)	97 (49.20)	80 (51.00)	17 (42.50)	0.437
Age	68.00 [60.00, 73.00]	68.00 [61.00, 73.00]	66.00 [57.00, 73.25]	0.464
Onset age	58.00 [51.00, 63.00]	59.00 [51.50, 64.00]	57.50 [49.50, 62.25]	0.447
Disease duration	5.98 [3.50, 8.97]	5.98 [3.50, 8.93]	5.66 [3.63, 9.33]	0.880
LED	150.00 [150.00, 200.00]	150.00 [150.00, 200.00]	150.00 [147.66, 200.00]	0.721
Drug type^a^, *n* (%)				0.377
0	164 (83.20)	131 (83.40)	33 (82.50)	
1	21 (10.70)	15 (9.60)	6 (15.00)	
2	12 (6.10)	11 (7.00)	1 (2.50)	
HY	3.00 [2.00, 3.00]	3.00 [2.00, 3.00]	3.00 [2.50, 3.00]	0.905
ΔDBP	0.00 [−6.00, 6.00]	1.00 [−6.00, 7.00]	0.00 [−3.25, 2.25]	0.816
ΔSBP	7.00 [0.00, 20.00]	7.00 [0.00, 23.00]	7.00 [1.75, 16.00]	0.695
Standing DBP	86.00 [77.00, 92.00]	85.00 [76.00, 91.00]	88.50 [78.00, 94.25]	0.292
Standing SBP	127.00 [112.00, 143.00]	127.00 [110.00, 141.00]	134.00 [118.75, 148.25]	0.073
Supine DBP	85.00 [78.00, 92.00]	84.00 [77.00, 91.00]	87.00 [79.75, 95.50]	0.172
Supine SBP	139.00 [126.00, 151.00]	138.00 [125.00, 150.00]	143.00 [129.75, 154.50]	0.144
OH, *n* (%)	56 (28.40)	50 (31.80)	6 (15.00)	0.056
MMSE	27.00 [24.00, 28.00]	27.00 [24.00, 28.00]	26.00 [23.00, 29.00]	0.730
MDS-UPDRS III (OFF)	39.00 [29.00, 54.00]	39.00 [27.00, 54.00]	39.50 [31.75, 49.25]	0.615
Tremor score (OFF)	7.00 [4.00, 10.00]	7.00 [4.00, 10.00]	6.50 [3.00, 10.00]	0.542
Rigidity score	8.00 [6.00, 11.00]	8.00 [5.00, 11.00]	9.50 [7.00, 11.00]	0.278
Hypokinesia score	15.00 [11.00, 23.00]	15.00 [11.00, 23.00]	17.00 [9.50, 23.25]	0.714
Postural instability score	5.00 [4.00, 9.00]	5.00 [3.00, 9.00]	5.50 [4.00, 9.25]	0.694
Midline score	10.00 [7.00, 14.00]	10.00 [7.00, 15.00]	10.00 [8.00, 14.00]	0.618
Resting tremor score	5.00 [2.00, 7.00]	5.00 [2.00, 7.00]	5.00 [1.00, 7.25]	0.840
Postural and kinetic tremors score	2.00 [1.00, 4.00]	2.00 [1.00, 4.00]	2.00 [0.75, 3.25]	0.275
Bradykinesia upper extremity score (MAS)	5.00 [3.00, 7.00]	5.00 [3.00, 7.00]	5.00 [3.00, 8.00]	0.873
Lower limb bradykinesia score	5.00 [3.00, 8.00]	5.00 [3.00, 8.00]	6.00 [4.00, 8.00]	0.425
Rigidity/tremor ratio	1.17 [0.67, 2.12]	1.08 [0.69, 2.00]	1.43 [0.66, 2.54]	0.183
MDS-UPDRS III/disease duration	7.15 [4.51, 10.57]	7.00 [4.62, 10.42]	7.27 [4.32, 11.43]	0.628
Tremor/disease duration	1.11 [0.66, 1.99]	1.16 [0.67, 1.99]	0.99 [0.50, 1.98]	0.421
Rigidity/disease duration	1.33 [0.78, 2.28]	1.29 [0.78, 2.10]	1.48 [0.77, 2.32]	0.340
Hypokinesia/disease duration	2.55 [1.64, 4.54]	2.55 [1.70, 4.26]	2.51 [1.49, 5.31]	0.777
Postural instability/disease duration	0.99 [0.60, 1.61]	0.99 [0.60, 1.61]	0.95 [0.62, 1.31]	0.907

Data are presented as mean (SD) for normally distributed continuous variables, median [Q1, Q3] for nonnormally distributed continuous variables, *n* (%) for categorical variables. ^a^Drug type: 0: Levodopa and Benserazide Hydrochloride Tablets; 1: Levodopa and Benserazide Hydrochloride Tablets and Carbidopa and Levodopa Sustained-release Tablets; 2: Carbidopa and Levodopa Sustained-release Tablets. ^*^p: Differences among training and test data were assessed using the chi-square test for categorical variables, the unpaired two-tailed t test for normally distributed continuous variables, and the Wilcoxon rank sum test for nonnormally distributed continuous variables. H-Y stage, Hoehn-Yahr stage; LED, levodopa equivalent dose; DBP, diastolic blood pressure; SBP, systolic blood pressure; OH, orthostatic hypotension; MMSE, Mini-Mental State Examination; MDS-UPDRS III, the Movement Disorder Society’s Unified Parkinson’s Disease Rating Scale III; MAS, most affected side.

### 3.2 Feature analysis between levodopa-responsive tremor and levodopa-resistant tremor

**Figure 1:**
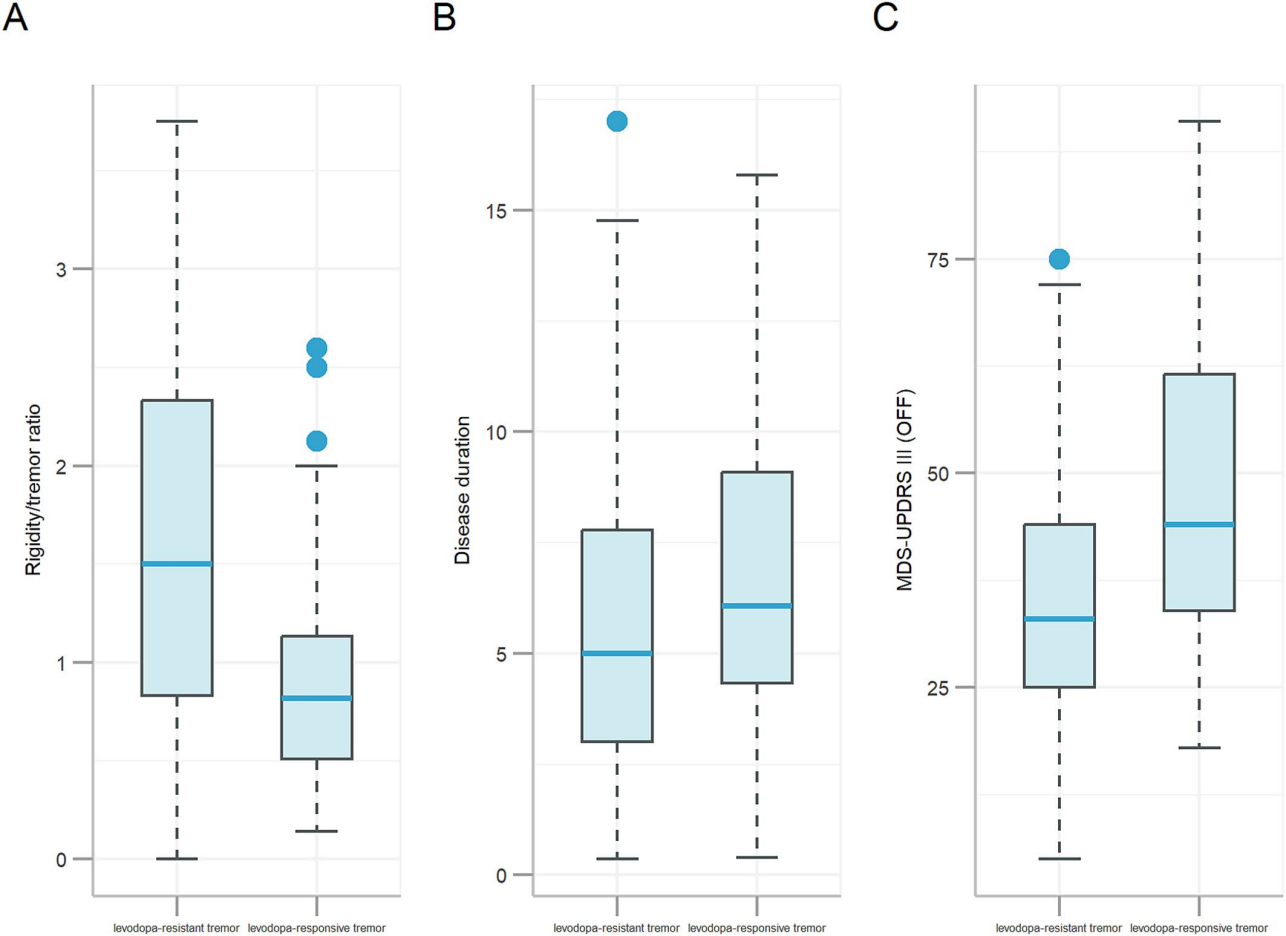
Boxplots of features: **(A)** Rigidity-to-tremor ratio, **(B)** Disease duration, and **(C)** MDS-UPDRS III score in the OFF-medication state. Each boxplot summarizes the data distribution as follows: The horizontal line within each box denotes the median. The top and bottom edges represent the 75th percentile (Q3) and 25th percentile (Q1). Whiskers extend to the maximum (Q3 + 1.5 × IQR) and minimum (Q1 − 1.5 × IQR) values, where IQR is the interquartile range. Outliers, defined as values beyond these whiskers, are shown as individual dots.

All features analyzed in this comparison were collected during the OFF-medication state, including the ‘Before LCT’ variables listed in Table 1 and EMG-derived features obtained prior to levodopa administration. In bivariate analysis, 14 features were significantly different (*p* < 0.05, highlighted in bold) between levodopa-responsive tremor and levodopa-resistant tremor groups (Table 4). For example, the Rigidity/tremor ratio of participants of levodopa-responsive tremor was 0.99 (*p* < 0.001, Figure 1A) lower than that of the participants of levodopa-resistant tremor on average. The participants of levodopa-responsive tremor had 1.08 years (*p* = 0.017, Figure 1B) longer disease duration than that of the participants of levodopa-resistant tremor on average. The MDS-UPDRS III (OFF) of participants of levodopa-responsive tremor was 11 (*p* < 0.001, Figure 1C) higher than that of the participants of levodopa-resistant tremor on average. Univariate ORs analysis was also shown in Table 4. For example, regarding disease duration (Univariate OR = 1.09, 95% CI: 1.01–1.20), the increase in odds of levodopa-responsive tremor occurrence was 9.0% for each unit (year) increase in disease duration without considering other factors. Regarding lower limb resting tremor frequency (MAS), compared to ‘0: Not seen,’ ‘1: Irregular jerks’ had an OR of 1.14 (95% CI: 0.40–3.20), indicating a non-significant increase in the odds of levodopa-responsive tremor occurrence. ‘2: <4’ had an OR of 1.42 (95% CI: 0.50–4.03), which was also not statistically significant. ‘4: >6’ had an OR of 2.85 (95% CI: 0.26–62.79), which was also not statistically significant. However, ‘3: [4, 6]’ had a statistically significant increase in the odds of levodopa-responsive tremor occurrence, with an OR of 3.50 (95% CI: 1.56–8.28), indicating that participants have lower limb resting tremor frequency (MAS) between 4 and 6 Hz have 3.5 times the odd of levodopa-responsive tremor occurrence compared to those whose lower limb resting tremor frequency (MAS) was not seen without considering other factors.

**Table 4:** Comparison of features between the levodopa-resistant tremor and levodopa-responsive tremor group on training data.

Feature	Levodopa-resistant tremor	Levodopa-responsive tremor	p*	Univariate OR (95% CI)
*n*	78	79		
Sex	38 (48.70)	42 (53.20)	0.691	1.19 (0.64, 2.24)
Age	68.00 [59.00, 72.75]	69.00 [63.00, 73.00]	0.306	1.02 (0.98, 1.05)
Onset age	58.00 [50.38, 64.75]	59.00 [52.00, 63.00]	0.813	0.99 (0.96, 1.02)
Disease duration	5.00 [3.00, 7.78]	6.08 [4.34, 9.30]	**0.017**	1.09 (1.01, 1.20)
HY	3.00 [2.00, 3.00]	3.00 [2.50, 3.00]	**0.013**	1.68 (1.18, 2.46)
Standing SBP	127.50 [112.75, 138.75]	127.00 [107.50, 145.00]	0.722	1.00 (0.98, 1.01)
Standing DBP	85.00 [79.00, 91.00]	86.00 [73.00, 91.50]	0.596	1.00 (0.98, 1.02)
Supine SBP	137.00 [124.25, 150.00]	139.00 [126.00, 151.00]	0.410	1.01 (0.99, 1.02)
Supine DBP	82.50 [78.25, 90.00]	85.00 [77.00, 92.50]	0.663	1.01 (0.99, 1.04)
ΔSBP	5.00 [−1.00, 18.50]	12.00 [2.50, 25.50]	**0.047**	1.01 (1.00, 1.03)
ΔDBP	−1.50 [−7.00, 5.75]	2.00 [−5.00, 8.00]	0.091	1.01 (0.99, 1.04)
OH	20 (25.6)	30 (38.0)	0.137	1.78 (0.9, 3.55)
MMSE	27.00 [24.25, 29.00]	27.00 [24.00, 28.00]	0.220	0.96 (0.88, 1.05)
LED	150.00 [150.00, 200.00]	200.00 [150.00, 200.00]	**0.020**	1.01 (1.00, 1.01)
Drug type			0.283	
0	63 (80.80)	68 (86.10)		Reference
1	7 (9.00)	8 (10.10)		1.06 (0.36, 3.18)
2	8 (10.30)	3 (3.80)		0.35 (0.07, 1.26)
MDS-UPDRS III (OFF)	33.00 [25.25, 44.00]	44.00 [34.00, 61.50]	**<0.001**	1.04 (1.02, 1.06)
Tremor score (OFF)	4.00 [2.00, 6.00]	10.00 [7.50, 13.00]	**<0.001**	1.59 (1.39, 1.87)
Resting tremor score	2.00 [0.00, 5.00]	7.00 [5.00, 9.00]	**<0.001**	1.64 (1.42, 1.93)
Postural and kinetic tremors score	2.00 [1.00, 2.75]	3.00 [1.00, 5.00]	**0.003**	1.36 (1.15, 1.62)
Rigidity score	7.00 [5.00, 10.00]	8.00 [6.00, 12.00]	0.102	1.06 (0.98, 1.15)
Hypokinesia score	14.00 [11.00, 18.00]	17.00 [11.00, 25.00]	0.056	1.04 (1.00, 1.07)
Postural instability score	5.00 [3.00, 8.00]	6.00 [4.00, 10.00]	0.070	1.08 (1, 1.16)
Midline score	9.00 [6.00, 13.00]	11.00 [8.00, 16.00]	**0.018**	1.06 (1.01, 1.12)
Bradykinesia upper extremity score (MAS)	5.00 [3.00, 6.75]	5.00 [4.00, 7.50]	0.320	1.08 (0.95, 1.22)
Lower limb bradykinesia score	4.00 [2.00, 7.00]	6.00 [4.00, 9.50]	**0.007**	1.11 (1.02, 1.22)
Rigidity/tremor ratio	1.81 [1.00, 3.46]	0.82 [0.51, 1.13]	**<0.001**	0.27 (0.15, 0.43)
MDS-UPDRS III/disease duration	7.08 [4.25, 11.16]	6.92 [5.21, 9.18]	0.731	1.00 (0.97, 1.03)
Tremor/disease duration	0.75 [0.40, 1.57]	1.65 [1.00, 2.27]	**<0.001**	1.53 (1.16, 2.1)
Rigidity/disease duration	1.37 [0.80, 2.66]	1.20 [0.74, 1.96]	0.237	0.96 (0.84, 1.08)
Hypokinesia/disease duration	2.72 [1.69, 4.90]	2.49 [1.88, 3.60]	0.415	0.98 (0.92, 1.04)
Postural instability/disease duration	1.01 [0.61, 1.67]	0.98 [0.61, 1.57]	0.637	1.01 (0.84, 1.23)
Upper limb resting tremor frequency (MAS)			0.215	
0: Not seen	36 (46.20)	23 (29.10)		Reference
1: Irregular jerks	17 (21.80)	18 (22.80)		1.66 (0.71, 3.89)
2: <4	2 (2.60)	2 (2.50)		1.57 (0.18, 13.8)
3: [4, 6]	20 (25.60)	32 (40.50)		2.50 (1.18, 5.46)
4: >6	3 (3.80)	4 (5.10)		2.09 (0.42, 11.42)
Upper limb resting tremor rhythm pattern (MAS)			0.218	
0: Not seen	36 (46.20)	23 (29.10)		Reference
1: Irregular jerks	17 (21.80)	18 (22.80)		1.66 (0.71, 3.89)
2: Alternation	11 (14.10)	17 (21.50)		2.42 (0.97, 6.22)
3: Synchronization	12 (15.40)	19 (24.10)		2.48 (1.03, 6.18)
4: Alternation + Synchronization	2 (2.60)	2 (2.50)		1.57 (0.18, 13.8)
Upper limb posture tremor frequency (MAS)			0.074	
0: Not seen	0 (0.00)	1 (1.30)		Reference
1: Irregular jerks	42 (53.80)	33 (41.80)		0.00 (NA, very large)^b^
2: <4	0 (0.00)	0 (0.00)		NA^c^
3: [4, 9]	33 (42.30)	45 (57.00)		0.00 (NA, very large)^b^
4: >9	3 (3.80)	0 (0.00)		0.00 (0.00, very large)^b^
Upper limb posture tremor rhythm pattern (MAS)			0.449	
0: Not seen	0 (0.00)	1 (1.30)		Reference
1: Irregular jerks	42 (53.80)	33 (41.80)		0.00 (NA, very large)^b^
2: Alternation	7 (9.00)	12 (15.20)		0.00 (NA, very large)^b^
3: Synchronization	26 (33.30)	29 (36.70)		0.00 (NA, very large)^b^
4: Alternation + Synchronization	3 (3.80)	4 (5.10)		0.00 (NA, very large)^b^
Upper limb kinetic tremor frequency (MAS)			0.088	
0: Not seen	1 (1.30)	3 (3.80)		Reference
1: Irregular jerks	45 (57.70)	31 (39.20)		0.23 (0.011, 1.887)
2: <4	0 (0.00)	0 (0.00)		NA^c^
3: [4, 9]	30 (38.50)	44 (55.70)		0.49 (0.02, 4.03)
4: >9	2 (2.60)	1 (1.30)		0.17 (0.00, 3.75)
Upper limb kinetic tremor rhythm pattern (MAS)			0.124	
0: Not seen	1 (1.30)	3 (3.80)		Reference
1: Irregular jerks	45 (57.70)	31 (39.20)		0.23 (0.01, 1.89)
2: Alternation	9 (11.50)	9 (11.40)		0.33 (0.01, 3.19)
3: Synchronization	22 (28.20)	32 (40.50)		0.48 (0.02, 4.07)
4: Alternation + Synchronization	1 (1.30)	4 (5.10)		1.33 (0.04, 44.45)
Upper limb holding condition tremor frequency (MAS)			0.339	
0: Not seen	1 (1.30)	2 (2.50)		Reference
1: Irregular jerks	38 (48.70)	33 (41.80)		0.43 (0.02, 4.73)
2: <4	1 (1.30)	0 (0.00)		0.00 (NA, very large)^b^
3: [4, 9]	34 (43.60)	43 (54.40)		0.63 (0.03, 6.87)
4: >9	4 (5.10)	1 (1.30)		0.12 (0.00, 2.62)
Upper limb holding condition tremor rhythm pattern (MAS)			0.799	
0: Not seen	1 (1.30)	2 (2.50)		Reference
1: Irregular jerks	38 (48.70)	33 (41.80)		0.43 (0.02, 4.73)
2: Alternation	10 (12.80)	8 (10.10)		0.40 (0.02, 4.93)
3: Synchronization	25 (32.10)	31 (39.20)		0.62 (0.03, 6.84)
4: Alternation + Synchronization	4 (5.10)	5 (6.30)		0.63 (0.02, 9.16)
Lower limb resting tremor frequency (MAS)			**0.045**	
0: Not seen	47 (60.30)	33 (41.80)		Reference
1: Irregular jerks	10 (12.80)	8 (10.10)		1.14 (0.40, 3.20)
2: <4	9 (11.50)	9 (11.40)		1.42 (0.50, 4.03)
3: [4, 6]	11 (14.10)	27 (34.20)		3.50 (1.56, 8.28)
4: >6	1 (1.30)	2 (2.50)		2.85 (0.26, 62.79)
Lower limb resting tremor rhythm pattern (MAS)			**0.043**	
0: Not seen	47 (60.30)	33 (41.80)		Reference
1: Irregular jerks	10 (12.80)	8 (10.10)		1.14 (0.40, 3.2)
2: Alternation	10 (12.80)	14 (17.70)		1.99 (0.80, 5.15)
3: Synchronization	11 (14.10)	24 (30.40)		3.11 (1.37, 7.43)
4: Alternation + Synchronization	0 (0.00)	0 (0.00)		NA^c^
Lower limb posture tremor frequency (MAS)			0.089	
0: Not seen	4 (5.10)	3 (3.80)		Reference
1: Irregular jerks	30 (38.50)	17 (21.50)		0.76 (0.15, 4.21)
2: <4	2 (2.60)	5 (6.30)		3.33 (0.39, 37.63)
3: [4, 9]	42 (53.80)	54 (68.40)		1.71 (0.36, 9.1)
4: >9	0 (0.00)	0 (0.00)		NA^c^
Lower limb posture tremor rhythm pattern (MAS)			0.119	
0: Not seen	4 (5.10)	3 (3.80)		Reference
1: Irregular jerks	30 (38.50)	17 (21.50)		0.76 (0.15, 4.21)
2: Alternation	7 (9.00)	11 (13.90)		2.10 (0.36, 13.58)
3: Synchronization	36 (46.20)	48 (60.80)		1.78 (0.37, 9.5)
4: Alternation + Synchronization	1 (1.30)	0 (0.00)		0.00 (NA, very large)^b^

Data are presented as mean (SD) for normally distributed continuous variables, median [Q1, Q3] for nonnormally distributed continuous variables, *n* (%) for categorical variables. ^a^Drug type: 0: Levodopa and Benserazide Hydrochloride Tablets; 1: Levodopa and Benserazide Hydrochloride Tablets and Carbidopa and Levodopa Sustained-release Tablets; 2: Carbidopa and Levodopa Sustained-release Tablets. ^b^(NA, very large), (0.00, very large): OR and 95% CI could not be reliably estimated due to complete separation or imbalanced data in this category. ^c^NA: Odds ratio not available (no participants in this category). ^*^p less than 0.05 is highlighted in bold, indicating a significant difference between the two groups. LED, levodopa equivalent dose; DBP, diastolic blood pressure; SBP, systolic blood pressure; OH, orthostatic hypotension; MMSE, Mini-Mental State Examination; MDS-UPDRS III, the Movement Disorder Society’s Unified Parkinson’s Disease Rating Scale III; MAS, most affected side.

### 3.3 Comparison of model performance

**Figure 2:**
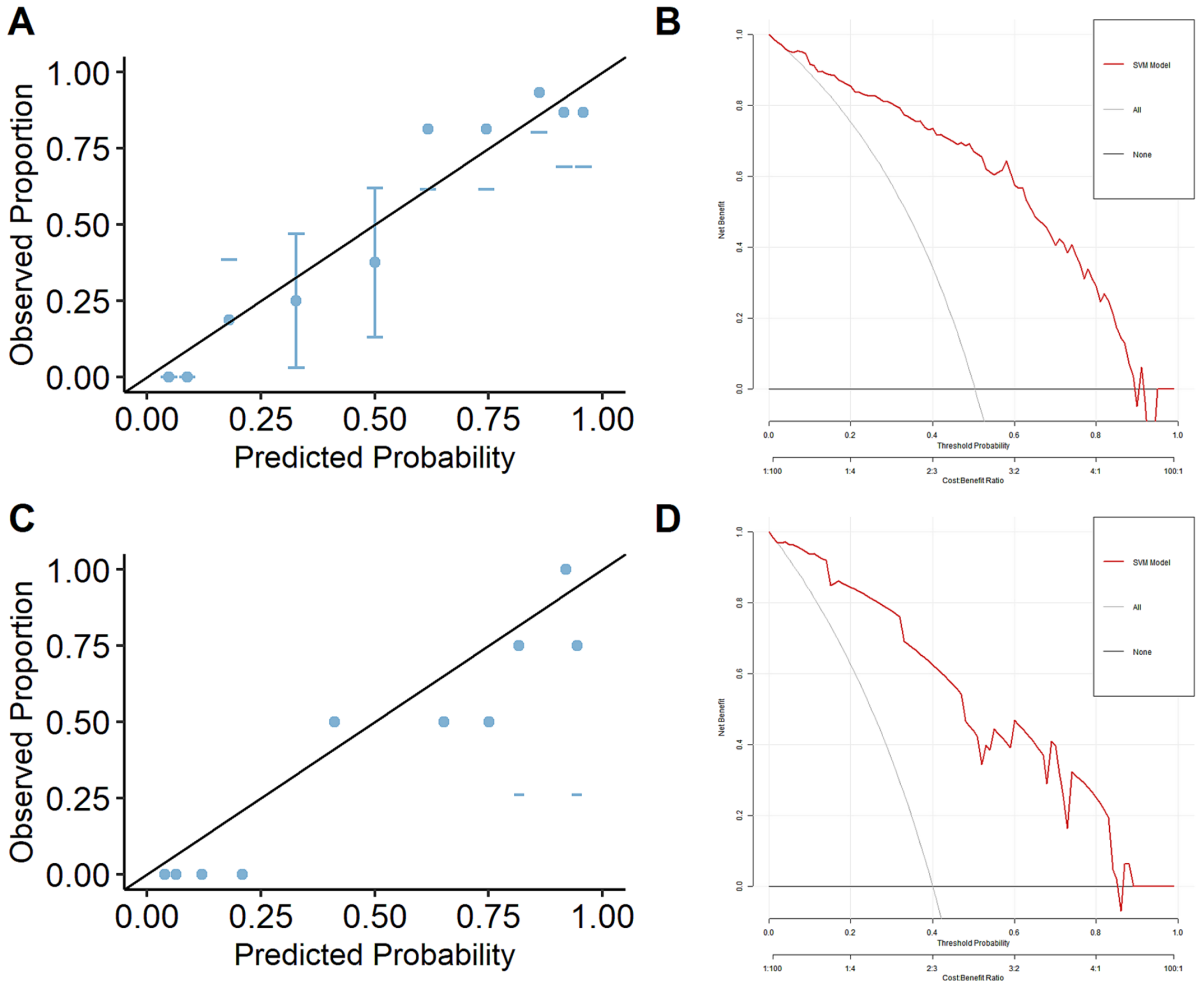
Calibration plot and decision curve of the final model. **(A)** Calibration plot on train data, **(B)** decision curve on train data, **(C)** calibration plot on test data, **(D)** decision curve on test data.

The model performance comparisons based on five-fold cross-validation on training data are shown in Table 5. SVM [ACC: 81.5%, Kappa 0.624, Sen: 84.3%, Spe: 77.9%, PPV: 80.2%, NPV: 84.4%, AUC: 0.850] outperformed the other two models, which shows good discrimination ability. Calibration plot shows good alignment between observed proportion of levodopa-responsive tremor cases and predicted probability of levodopa-responsive tremor cases obtained from the best model (SVM) on training data as the points were around the 45-degree line, which indicates a good calibration ability (Figure 2A). Decision curve shows the decision curve of the best model (SVM) on training data (Figure 2B). We can see that, except for a small range of high threshold probability, intervening on patients based on the best prediction model (SVM) of our study leads to higher net benefit than intervention for all or intervention for none. Therefore, we chose this model as the final model in our study. To further evaluate model robustness and determine whether the observed performance differences among the three classifiers were statistically meaningful, we conducted an additional repeated 5-fold cross-validation (20 repetitions; 100 resamples). Across the resamples, the mean accuracies were comparable among SVM (0.785 ± 0.069; 95% CI: 0.650–0.923), RF (0.770 ± 0.062; 95% CI: 0.646–0.875), and LR (0.784 ± 0.059; 95% CI: 0.677–0.875). Paired t-tests and Wilcoxon signed-rank tests revealed no statistically significant differences among the three models (all *p* > 0.05). Despite the lack of statistically significant differences, the SVM consistently showed the highest average accuracy across resamples and required fewer features compared with RF and LR. These findings further support the choice of SVM as the final predictive model, in line with the primary cross-validation results.

**Table 5:** Model performance on five-fold cross-validation.

Classifier	ACC	Kappa	Sen	Spe	ppv	npv	AUC	fea_num	Hyperparameter
RF	78.4%	0.563	76.1%	80.5%	81.5%	78.4%	0.838 [0.693, 0.981]	8	mtry:1, nodesize:5, ntree:200
SVM	81.5%	0.624	84.3%	77.9%	80.2%	84.4%	0.850 [0.701, 0.990]	6	C:0.55, sigma:0.06
LR	77.7%	0.543	73.8%	80.1%	79.8%	77.0%	0.834 [0.685, 0.981]	8	/

ACC, accuracy; Sen, sensitivity; Spe, specificity; ppv, positive predictive value; npv, negative predictive value; AUC_ROC, receiver operating characteristic area under the curve.

### 3.4 Independent test data validation

The final prediction model (SVM) was validated on the independent test set. The model performance on the independent test data [ACC: 82.5%, Kappa 0.653, Sen:93.8%, Spe: 75%, PPV: 71.4%, NPV: 94.7%, AUC: 0.896 (95% CI: 0.799, 0.992)] was as good as the five-fold cross validation model performance on training data, which indicated a good reproducibility of our model. In our study, this final model was able to correctly classify 82.5% patients as levodopa-responsive tremor and levodopa-resistant tremor, correctly classify 93.8% patients as levodopa-responsive tremor out of all levodopa-responsive tremor, and correctly classify 75% patients as levodopa-resistant tremor out of all levodopa-resistant tremor. The probability that a patient was predicted as levodopa-responsive tremor actually was 71.4%, while the probability that a participant predicted as levodopa-resistant tremor truly was levodopa-resistant tremor was 94.7%. Calibration plot demonstrates good alignment between observed proportion of levodopa-responsive tremor cases and predicted probability of levodopa-responsive tremor cases obtained from this final model on the test data, as indicated by the points closely aligning with the 45-degree line, reflecting good calibration (Figure 2C). The decision curve presents for this final model on the test data (Figure 2D). It shows that, except for a small range of high threshold probabilities, making intervention decisions based on the final prediction model yields a higher net benefit compared to intervening on all patients or none, which concludes that using this prediction model to determine whether patients should receive intervention would lead to good clinical outcome.

### 3.5 Predictors for optimal predictive model

**Figure 3:**
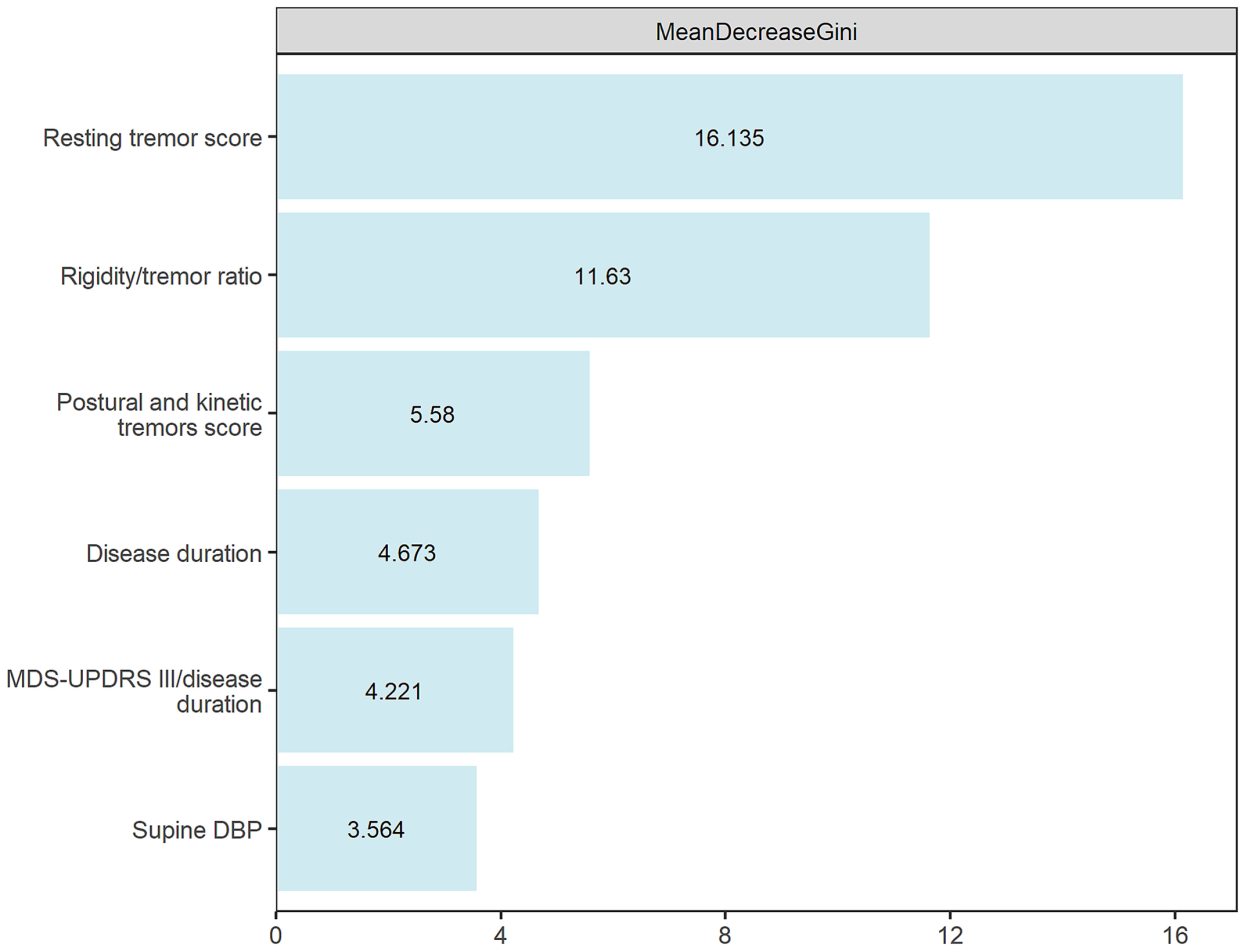
MD Gini of features in the final predictive model construction.

The MD Gini for the predictors included in our optimal predictive model (SVM) were presented in Figure 3. A total of 6 predictors were incorporated into the final model with hyperparameters C as 0.55 and sigma as 0.06. Resting tremor score demonstrated the highest MD Gini, indicating it as the most important predictor in the construction of our predictive model. The remaining 5 predictors included rigidity/tremor ratio, postural and kinetic tremors score, disease duration, MDS-UPDRS III/disease duration, and supine DBP.

## 4 Discussion

Our study is the first to predict the responsiveness of PD tremor to levodopa treatment using machine learning models. Among the models evaluated, the SVM achieved the highest predictive performance and successfully passed validation, followed by RF and LR. The final SVM model incorporated 6 key predictors and demonstrated robust performance, offering a potential foundation for personalized treatment of tremor in PD patients. The selected predictors included: resting tremor score, rigidity/tremor ratio, postural and kinetic tremors score, disease duration, MDS-UPDRS III/disease duration, and supine DBP. The SVM model was trained with optimized hyperparameters, with the regularization parameter C set to 0.55 and sigma (kernel width) set to 0.06. Although the independent test accuracy of our final SVM model was 0.825, this level of performance is consistent with previous machine-learning studies addressing similar clinical prediction tasks in PD. For example, a machine-learning model predicting acute orthostatic hypotension after levodopa administration achieved a leave-one-out cross-validation (LOOCV) accuracy of 73.6% and an independent test accuracy of 72% (Liu et al., 2024). Likewise, wearable sensor–based gait-classification models distinguishing early PD from essential tremor reported LOOCV accuracy of 84% and independent test accuracy of 75.8% (Lin et al., 2023). These findings indicate that predictive accuracies around 0.75–0.80 are typical and considered acceptable for PD-related machine-learning models that aim to support rather than replace clinical decision-making. Therefore, the performance achieved by our model (test accuracy 0.825) falls within the expected and clinically meaningful range.

Recently, machine learning techniques have been increasingly utilized to improve the prediction of complex and hard-to-predict outcomes. SVM, RF, and LR were utilized to predict the responsiveness of PD tremor in our study. Our results show that SVM and RF performed better than the traditional machine learning method, LR. Traditional machine learning models were typically suitable for linearly separable data, under the assumption that the features exhibit linear relationships, independence, and specific distribution characteristics, which may not hold in real-world datasets (Peng et al., 2002). However, real-world medical data often present with high non-linearity and complex feature interactions, which limit the expressive power of LR in such settings. Compared with LR, other machine learning algorithms, such as SVM and RF, possess greater flexibility and modeling capacity, which do not rely on predefined functional forms and are capable of capturing complex non-linear relationships and feature interactions (Deo, 2015). Therefore, machine learning offers a powerful and flexible framework for developing predictive models, especially when dealing with complex, noisy, or high-dimensional data that may violate traditional modeling assumptions.

Although neural-network–based models have gained increasing attention in biomedical classification tasks (Huang et al., 2021; Martinez-Murcia et al., 2021), their application typically requires substantially larger datasets and offers limited interpretability, which may hinder their clinical usefulness. Given the modest sample size of the present cohort and the importance of transparent predictive features, classical machine-learning algorithms were prioritized. To further examine whether a more complex non-linear model could provide additional benefit, we conducted an exploratory analysis using a regularized multilayer perceptron neural-network classifier, applying the same feature-selection strategy as in the main analysis. The neural-network model achieved cross-validated performance [ACC: 73.8%, Kappa 0.472, Sen: 83.4%, Spe: 63.9%, PPV: 71.5%, NPV: 80.7%, AUC: 0.777] that was comparable to, but not superior to, that of the SVM, RF, and LR models. These findings suggest that deep-learning approaches did not offer measurable advantages under the current study conditions and support the appropriateness of selecting classical machine-learning algorithms for the final model development.

In this study, the SVM model was selected as our final model as it outperformed both the RF and LR models. SVM constructs a maximum-margin hyperplane and leverages kernel functions (such as the Radial Basis Function, RBF) to map low-dimensional non-linear problems into high-dimensional space, thereby enabling efficient classification of complex data structures (Noble, 2006). This characteristic makes SVM particularly suitable for our dataset, which is characterized by non-linear and partially overlapping clinical and EMG feature distributions within a moderate sample size. In our study, the SVM model was configured with two key hyperparameters: C = 0.55 and sigma = 0.06. The parameter C is the regularization parameter in SVM, which controls the trade-off between maximizing the margin (generalization) and minimizing classification errors (especially for noisy or overlapping data). A smaller C (e.g., 0.55) allows the model to tolerate some degree of misclassification, thus helping to avoid overfitting and improve generalization. The parameter sigma (the bandwidth of the RBF kernel) defines the spread of the kernel, influencing how far the influence of a single training example reaches. A sigma = 0.06 means the kernel is narrow, making the model highly sensitive to nearby points. This can capture complex, non-linear patterns but risks overfitting if the data is noisy. The combined impact of C = 0.55 and sigma = 0.06 corresponded to a conservative model: The small C suggests a preference for simplicity (larger margin), while the small sigma allows flexibility in non-linear decisions. In conclusion, SVM offers theoretical advantages in boundary control and non-linear modeling over LR and RF. With appropriately tuned hyperparameters, it further improves its performance in predicting clinical tremor responsiveness.

**Figure 4:**
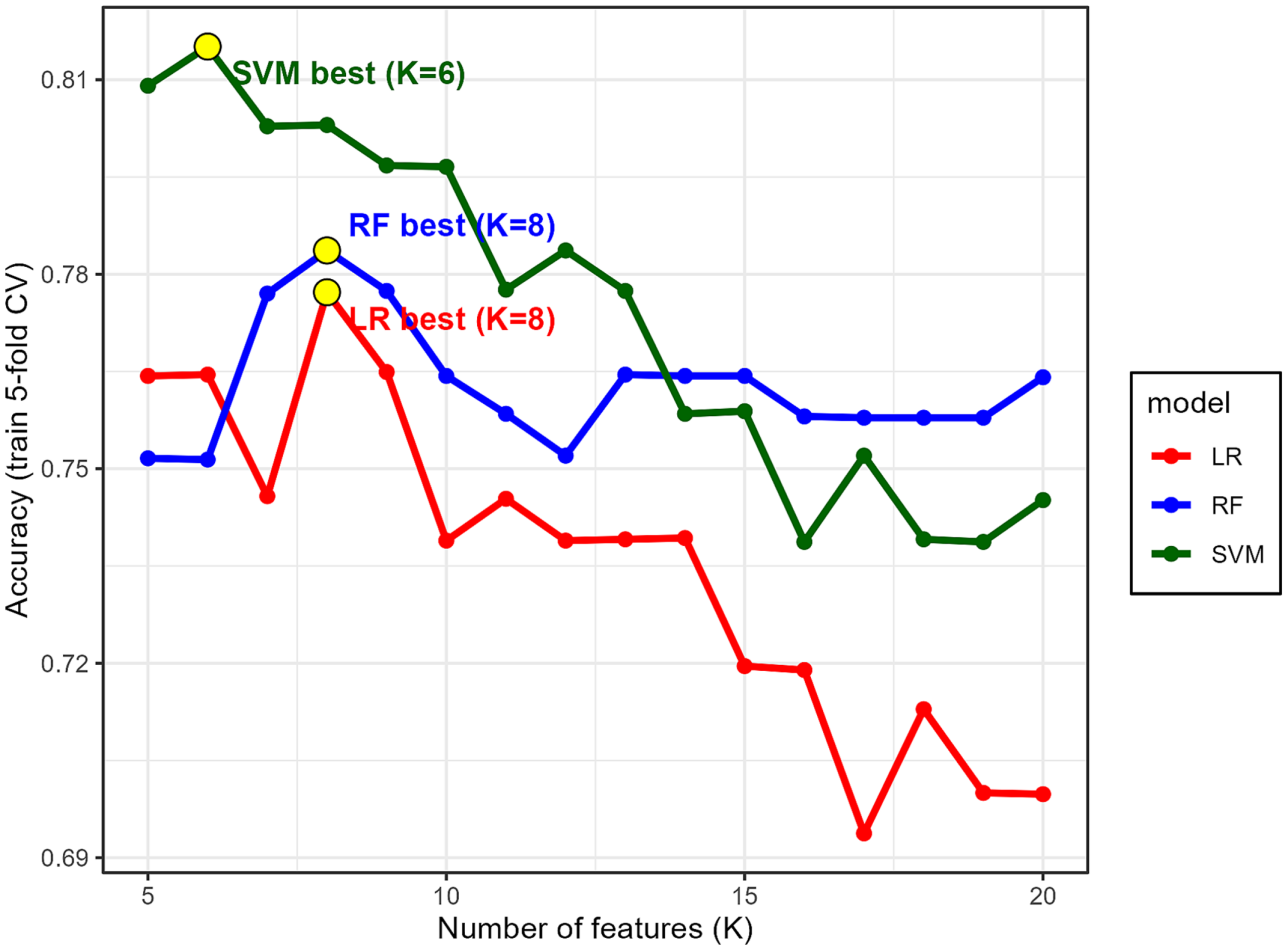
Cross-validated accuracy of SVM, RF, and LR across different numbers of selected features (K).

In addition to identifying the optimal model, we further examined whether the six predictors selected by the SVM reflect a generalizable subset of informative features across different classifiers. All three models (SVM, RF, and LR) were trained using feature subsets derived from the same Random Forest permutation-importance ranking, ensuring full comparability. Although the optimal number of predictors differed slightly among models (SVM: *K* = 6; RF and LR: *K* = 8), the selected feature sets showed substantial overlap, indicating that the most influential predictors were largely shared across classifiers rather than being specific to a single method. To evaluate how sensitive model performance was to the number of predictors, we visualized the cross-validated accuracy of each classifier across different values of *K* using a new summary Figure 4. These curves were generated directly from the results of our original modeling pipeline, in which each classifier had already been trained and fully hyperparameter-tuned at every value of K (as described in the Methods section). For each *K*, the plotted accuracy represents the best-performing hyperparameter configuration—that is, the highest cross-validated accuracy obtained among all tested hyperparameter combinations. As shown in Figure 4, SVM achieved its peak accuracy at *K* = 6 and remained stable across *K* = 5–10; RF performed best at *K* = 8 with stable performance across *K* = 7–10; and LR also peaked at *K* = 8, followed by a gradual decline thereafter. These smoothly varying performance patterns indicate that none of the classifiers are overly sensitive to small changes in the number of predictors.

Having established the performance and configuration of the SVM model, we then explored the clinical relevance of the selected predictors. In this study, the key discriminative indices of tremor responsiveness to levodopa based on SVM were discussed while considering demographic factors, clinical data, evaluations of motor symptoms, and evaluations of non-motor symptoms. The prominent predictors of our final prediction model included the resting tremor score, postural and kinetic tremor score, and disease duration. They were also the most important features for discriminating levodopa-responsive tremor from the levodopa-resistant tremor group based on univariate analysis. We found that the levodopa-responsive tremor group had higher tremor severity and longer disease duration, which were similar to previous studies. Imaging studies demonstrate that resting tremor is usually associated with a dopaminergic deficit (Brooks et al., 1992). However, tremor severity does not correlate with striatum dopamine depletion in other studies (Louis et al., 2001; Pirker, 2003). This suggests that tremor in PD can be understood merely as an expression of dopaminergic denervation of the basal ganglia, but that other neurotransmitter systems and brain areas are involved (Dirkx and Bologna, 2022). Thus, tremors in PD may respond differently to dopaminergic replacement therapy. Our results further supported the conclusion that a significant response to levodopa was still observed in patients with more pronounced resting tremor as well as postural and kinetic tremor, and those with longer disease duration (Swinnen et al., 2025). However, given that the mean disease duration of the patients in this study was 5.98 years and the mean tremor score was 7 points, it remains to be investigated whether PD patients with a longer disease duration and a more severe tremor score than the average in our study have a different levodopa response. In our study, we found that tremor score and disease duration had a significant correlation in the responsiveness of PD tremor to levodopa. Therefore, the resting tremor score, postural and kinetic tremor score, and disease duration were found to be the key predictors rationally.

Although tremors were more pronounced in the levodopa-responsive tremor group, their progression patterns were discordant. Our study also found a faster rate of tremor progression in the levodopa-responsive tremor group, with no difference in the progression of the other motor symptoms and overall rate of motor progression between the two groups. The levodopa-resistant tremor group had a higher rigidity/tremor ratio than the levodopa-responsive tremor group through multivariate analysis. Tremors progressed at their own pace, and tremor severity did not correlate with the severity of bradykinesia or rigidity (Louis et al., 2001). This reflects that there may be a unique mechanism for tremor compared to other PD motor symptoms, such as rigidity. Except for multifactorial interactions, the rigidity/tremor ratio and MDS-UPDRS III/disease duration ratio were the contributions and valid predictors for our model construction.

In this study, supine DBP before medication was retained for our SVM model construction, providing new insights into the potential involvement of autonomic dysfunction in PD tremor. Comparison between the levodopa-resistant tremor and levodopa-responsive tremor groups revealed a statistical difference in ΔSBP; whereas standing SBP, supine SBP, standing DBP, supine DBP, ΔDBP, and OH show no significant differences. Although supine DBP did not differ significantly between groups, its retention in the model suggests potential relevance when combined with other predictors. This finding should nevertheless be interpreted with caution, as supine DBP was one of six potential predictors in our final model and was not consistently significant in univariate analyses. This suggests that its contribution should therefore be interpreted in conjunction with the other predictors in the model. Previous studies have shown that levodopa is well known for its hypotensive effects, which can lead to OH in PD patients through both peripheral and central mechanisms (Kujawa et al., 2000; Palma et al., 2015; Liu et al., 2024). Recent evidence suggested that PD patients who had a better response to levodopa were likely to have a greater impact of the hypotensive effect of levodopa. Additionally, PD is a primary cause of autonomic failure, with OH often occurring even in the prodromal stage and in drug-naive patients (Postuma et al., 2013; Dommershuijsen et al., 2021). Given these observations, the presence of supine DBP in our final model may reflect an underlying autonomic component influencing the dopaminergic responsiveness of tremor. This finding highlights the importance of incorporating autonomic parameters into future predictive and mechanistic studies on PD tremor.

By comparing groups, we found that EMG features may reflect differences in tremor subtypes in PD, with a higher proportion of irregular jerks in the levodopa-resistant tremor group and more 4–6 Hz classical tremor in the levodopa-responsive group. One possible explanation for these findings is that voluntary movement increases neural excitability within the cerebello-thalamo-cortical motor circuit, resulting in faster (higher frequency) synaptic transmission and making it less dependent on dopaminergic influences from the basal ganglia (reduced dopamine response) (Dirkx et al., 2016). Although these findings suggest physiological heterogeneity between subtypes, none of the EMG-derived features were retained in the final SVM model. This indicates that their discriminative information likely overlapped with other clinical predictors, and their incremental contribution to the model’s performance was limited. Therefore, EMG characteristics may not provide additional predictive value beyond the core clinical features, which serve as more stable and clinically practical indicators.

Furthermore, our findings suggest that relatively simple and routinely available clinical features, such as resting tremor score, rigidity/tremor ratio, disease duration, and supine DBP, provided stronger predictive performance than complex EMG-derived features. This highlights the potential for developing clinically practical models that rely primarily on accessible clinical assessments rather than specialized electrophysiological data. The development of these pragmatic tools is therefore crucial for broadening access to precision medicine. This includes access to clinical decision workflows that help personalize therapeutic strategies, particularly in resource-limited environments.

Our findings indicate that several clinical features assessed during the OFF-medication state, including resting tremor score, rigidity/tremor ratio, postural and kinetic tremors score, disease duration, MDS-UPDRS III/disease duration, supine DBP, demonstrated significant discriminative power in predicting tremor responsiveness to the levodopa challenge. These results underscore the potential of these OFF-state markers as accessible, non-invasive predictors for stratifying patients based on their likelihood of benefiting from dopaminergic therapy. Emerging evidence suggests that the distinct disease trajectories, often categorized as tremor-dominant (TD) and postural instability/gait difficulty (PIGD) subtypes. These clinical findings may have their underpinnings in the recently proposed body-first and brain-first hypotheses of disease propagation (Dirkx et al., 2018). It has also been shown that data-driven subtyping, rather than clinical or pathological subtyping, provides a promising strategy to inform disease modification trials in PD (Negida et al., 2025). To operationalize these predictors, we developed an SVM classification model, which showed robust performance in distinguishing between levodopa-responsive and levodopa-resistant tremor subtypes based on the selected OFF-state features. Integrating such a machine learning model into clinical workflows could enhance decision-making by providing objective, data-driven support for individualizing treatment strategies, particularly in cases where tremor responsiveness remains uncertain. Further research is warranted to validate both the predictors and the model in larger, prospective cohorts and to explore their implementation in practical clinical tools such as clinical decision support systems or digital health platforms.

This study has several limitations. First, although clinical and demographic characteristics were thoroughly analyzed, the EMG characteristics were excluded from the predictive model as their contribution was not statistically significant. Incorporation of machine learning models based on more comprehensive EMG characteristics, MRI, and other biomarkers could potentially improve classification accuracy. Future studies with multidimensional data are needed. Second, machine learning has the advantage of leveraging training set resources to their fullest through intelligent algorithms, ensuring the efficiency of the resulting model. However, a major challenge is the potential for overfitting. Therefore, it is essential to construct an efficient and robust machine learning model using multi-center, multi-dimensional, and large datasets in the future. Third, there is no consistent definition of levodopa responsiveness for tremor in PD across various studies currently. Some studies have classified patients based on the median tremor change rate score (Wu et al., 2022; Zhang et al., 2022). Levodopa tremor responsiveness was calculated as the difference in tremor score in another study (Sung et al., 2008). Using cluster analysis, we optimized the cutoff value by maximizing the AUC to ensure the best classification performance. Fourth, both the training and test sets were derived from the same population, and additional validation in new patients from a different but related population is essential. Fifth, although the dataset in this study was relatively balanced (95 levodopa-responsive tremor vs. 102 levodopa-resistant tremor patients), minor class imbalance could still have a subtle effect on model learning. Because both the training and test sets maintained comparable proportions of each class, resampling or class-weighting methods were not applied. Future studies with larger and more heterogeneous datasets should further validate model stability under different class distributions. Sixth, certain subgroups had relatively small sample counts, such as the lower-limb resting tremor frequency categories (e.g., “>6 Hz”). This limited the stability and precision of the corresponding odds ratio estimates, as reflected by wide confidence intervals (e.g., OR = 2.85, 95% CI: 0.26–62.79). These sparse data increase the uncertainty of statistical inference and raise potential risks of both Type I (false-positive) and Type II (false-negative) errors. Therefore, these findings should be interpreted with caution, and validation in larger samples is warranted. Seventh, we did not investigate the subtypes of PD patients or tremor types based on levodopa response. Future studies with larger sample sizes are needed to investigate the prognosis of different subtypes. Finally, the study did not evaluate the long-term levodopa responsiveness of tremor in PD patients, suggesting the need for further longitudinal research. Despite these limitations, our study offers novel insights and a practical framework for integrating predictive modeling into tremor management in Parkinson’s disease.

## 5 Conclusion

In conclusion, the construction of machine learning models from different perspectives based on demographic and clinical indicators has high predictive efficiency and clinical net benefit for levodopa response of tremor in PD. The features used to construct our final predictive model are expected to serve as potential predictive markers for the responsiveness of PD tremor to levodopa, further deepening our understanding of its pathogenesis. Our SVM model may become an effective and clinically applicable new method to provide a basis for the precise treatment of tremor in PD patients.

## Data Availability

The raw data supporting the conclusions of this article will be made available by the authors without undue reservation.

## References

[ref1] Albanese A. Bonuccelli U. Brefel C. Chaudhuri K. R. Colosimo C. Eichhorn T. . (2001). Consensus statement on the role of acute dopaminergic challenge in Parkinson's disease. Mov. Disord. 16, 197–201. doi: 10.1002/mds.1069, 11295770

[ref2] Breiman L. J. (2001). Random forests. Mach. Learn. 45, 5–32. doi: 10.1023/A:1010933404324

[ref3] Brooks D. J. Playford E. D. Ibanez V. Sawle G. V. Thompson P. D. Findley L. J. . (1992). Isolated tremor and disruption of the nigrostriatal dopaminergic system: an 18F-dopa PET study. Neurology 42:1554. doi: 10.1212/wnl.42.8.1554, 1641153

[ref4] Deo R. C. (2015). Machine learning in medicine. Circulation 132, 1920–1930. doi: 10.1161/CIRCULATIONAHA.115.001593 26572668 PMC5831252

[ref5] Dirkx M. F. Bologna M. (2022). The pathophysiology of Parkinson's disease tremor. J. Neurol. Sci. 435:120196. doi: 10.1016/j.jns.2022.120196, 35240491

[ref6] Dirkx M. F. den Ouden H. Aarts E. Timmer M. Bloem B. R. Toni I. . (2016). The cerebral network of Parkinson's tremor: an effective connectivity fMRI study. J. Neurosci. 36, 5362–5372. doi: 10.1523/jneurosci.3634-15.2016, 27170132 PMC6601802

[ref7] Dirkx M. F. Zach H. Bloem B. R. Hallett M. Helmich R. C. (2018). The nature of postural tremor in Parkinson disease. Neurology 90, e1095–e1103. doi: 10.1212/wnl.0000000000005215, 29476038 PMC5880634

[ref8] Dirkx M. F. Zach H. van Nuland A. Bloem B. R. Toni I. Helmich R. C. (2019). Cerebral differences between dopamine-resistant and dopamine-responsive Parkinson's tremor. Brain 142, 3144–3157. doi: 10.1093/brain/awz261, 31509182

[ref9] Dommershuijsen L. J. Heshmatollah A. Mattace Raso F. U. S. Koudstaal P. J. Ikram M. A. Ikram M. K. (2021). Orthostatic hypotension: a prodromal marker of Parkinson's disease? Mov. Disord. 36, 164–170. doi: 10.1002/mds.28303, 32965064 PMC7891584

[ref10] Feng T. Li W. Lu L. Wang Y. Shi W. Zhang J. . (2009). Acute stepwise challenge test with levodopa in treated patients with Parkinsonism. Parkinsonism Relat. Disord. 15, 354–358. doi: 10.1016/j.parkreldis.2008.08.010, 19010079

[ref11] Fishman P. S. (2008). Paradoxical aspects of parkinsonian tremor. Mov. Disord. 23, 168–173. doi: 10.1002/mds.21736, 17973325

[ref12] Freeman R. Wieling W. Axelrod F. B. Benditt D. G. Benarroch E. Biaggioni I. . (2011). Consensus statement on the definition of orthostatic hypotension, neurally mediated syncope and the postural tachycardia syndrome. Clin. Auton. Res. 21, 69–72. doi: 10.1007/s10286-011-0119-5, 21431947

[ref13] Goetz C. G. Tilley B. C. Shaftman S. R. Stebbins G. T. Fahn S. Martinez-Martin P. . (2008). Movement Disorder Society-sponsored revision of the Unified Parkinson's Disease Rating Scale (MDS-UPDRS): scale presentation and clinimetric testing results. Mov. Disord. 23, 2129–2170. doi: 10.1002/mds.22340, 19025984

[ref14] Helmich R. C. Hallett M. Deuschl G. Toni I. Bloem B. R. (2012). Cerebral causes and consequences of parkinsonian resting tremor: a tale of two circuits? Brain 135, 3206–3226. doi: 10.1093/brain/aws023, 22382359 PMC3501966

[ref15] Huang Z. Sun M. Guo C. (2021). Automatic diagnosis of Alzheimer’s disease and mild cognitive impairment based on CNN+ SVM networks with end-to-end training. Comput. Intell. Neurosci. 2021:9121770. doi: 10.1155/2021/9121770, 34426737 PMC8380157

[ref16] Kujawa K. Leurgans S. Raman R. Blasucci L. Goetz C. G. (2000). Acute orthostatic hypotension when starting dopamine agonists in Parkinson's disease. Arch. Neurol. 57, 1461–1463. doi: 10.1001/archneur.57.10.1461, 11030798

[ref17] Lin S. Gao C. Li H. Huang P. Ling Y. Chen Z. . (2023). Wearable sensor-based gait analysis to discriminate early Parkinson’s disease from essential tremor. J. Neurol. 270, 2283–2301. doi: 10.1007/s00415-023-11577-6, 36725698 PMC10025195

[ref18] Liu Z. Lin S. Zhou J. Wang X. Wang Z. Yang Y. . (2024). Machine-learning model for the prediction of acute orthostatic hypotension after levodopa administration. CNS Neurosci. Ther. 30:e14575. doi: 10.1111/cns.14575, 38467597 PMC10927600

[ref19] Louis E. D. Levy G. Côte L. J. Mejia H. Fahn S. Marder K. (2001). Clinical correlates of action tremor in Parkinson disease. Arch. Neurol. 58, 1630–1634. doi: 10.1001/archneur.58.10.1630, 11594921

[ref20] Martinez-Murcia F. J. Ortiz A. Ramírez J. Górriz J. M. Cruz R. (2021). Deep residual transfer learning for automatic diagnosis and grading of diabetic retinopathy. Neurocomputing 452, 424–434. doi: 10.1016/j.neucom.2020.04.148

[ref21] Negida A. Mukhopadhyay N. Berman B. D. Barrett M. J. (2025). Comparative analysis of progression milestones across Parkinson’s disease clinical, pathological, and data-driven subtypes: a 10-year follow-up. Res. Sq. 23:rs.3.rs-6574563. doi: 10.21203/rs.3.rs-6574563/v1 PMC13616926 42286020

[ref22] Noble W. S. (2006). What is a support vector machine? Nat. Biotechnol. 24, 1565–1567. doi: 10.1038/nbt1206-1565 17160063

[ref23] Palma J. A. Gomez-Esteban J. C. Norcliffe-Kaufmann L. Martinez J. Tijero B. Berganzo K. . (2015). Orthostatic hypotension in Parkinson disease: how much you fall or how low you go? Mov. Disord. 30, 639–645. doi: 10.1002/mds.26079, 25678194 PMC4397106

[ref24] Peng C.-Y. J. Lee K. L. Ingersoll G. M. (2002). An introduction to logistic regression analysis and reporting. J. Educ. Res. 96, 3–14. doi: 10.1080/00220670209598786

[ref25] Pieterman M. Adams S. Jog M. (2018). Method of levodopa response calculation determines strength of association with clinical factors in Parkinson disease. Front. Neurol. 9:260. doi: 10.3389/fneur.2018.00260, 29867708 PMC5966537

[ref26] Pirker W. (2003). Correlation of dopamine transporter imaging with parkinsonian motor handicap: how close is it? Mov. Disord. 18, S43–S51. doi: 10.1002/mds.10579, 14531046

[ref27] Pirker W. Katzenschlager R. Hallett M. Poewe W. (2023). Pharmacological treatment of tremor in Parkinson's disease revisited. J. Parkinsons Dis. 13, 127–144. doi: 10.3233/jpd-225060, 36847017 PMC10041452

[ref28] Postuma R. B. Berg D. Stern M. Poewe W. Olanow C. W. Oertel W. . (2015). MDS clinical diagnostic criteria for Parkinson's disease. Mov. Disord. 30, 1591–1601. doi: 10.1002/mds.26424, 26474316

[ref29] Postuma R. B. Gagnon J. F. Pelletier A. Montplaisir J. (2013). Prodromal autonomic symptoms and signs in Parkinson's disease and dementia with Lewy bodies. Mov. Disord. 28, 597–604. doi: 10.1002/mds.25445, 23554107

[ref30] Solida A. Ghika J. Vingerhoets F. (2002). Acute dopaminergic challenge tests to assess postural/kinetic tremor of different origin: a case report. J. Neurol. Neurosurg. Psychiatry 73, 206–207. doi: 10.1136/jnnp.73.2.206, 12122189 PMC1737967

[ref31] Sung Y. H. Chung S. J. Kim S. R. Lee M. C. (2008). Factors predicting response to dopaminergic treatment for resting tremor of Parkinson's disease. Mov. Disord. 23, 137–140. doi: 10.1002/mds.21793, 17987649

[ref32] Swinnen B. Frequin H. L. Wiggerts Y. Espay A. J. Beudel M. de Bie R. M. A. (2025). Tremor is highly responsive to levodopa in advanced Parkinson's disease. Mov. Disord. Clin. Pract. 12, 76–81. doi: 10.1002/mdc3.14262, 39520315 PMC11736880

[ref33] Van Calster B. McLernon D. J. van Smeden M. Wynants L. Steyerberg E. W. (2019). Calibration: the Achilles heel of predictive analytics. BMC Med. 17:230. doi: 10.1186/s12916-019-1466-7, 31842878 PMC6912996

[ref34] Wu J. Zhou C. Guo T. Guan X. Gao T. Bai X. . (2022). Cholinergic relevant functional reactivity is associated with dopamine responsiveness of tremor in Parkinson's disease. Brain Imaging Behav. 16, 1234–1245. doi: 10.1007/s11682-021-00610-9, 34973120 PMC9107430

[ref35] Zach H. Dirkx M. F. Roth D. Pasman J. W. Bloem B. R. Helmich R. C. (2020). Dopamine-responsive and dopamine-resistant resting tremor in Parkinson disease. Neurology 95, e1461–e1470. doi: 10.1212/wnl.0000000000010316, 32651292

[ref36] Zhang Y. He X. Mo C. Liu X. Li J. Yan Z. . (2022). Association between microbial tyrosine decarboxylase gene and Levodopa responsiveness in patients with Parkinson disease. Neurology 99, e2443–e2453. doi: 10.1212/wnl.0000000000201204, 36240098

[ref37] Zhu X. Chen Z. Ling Y. Luo N. Yin Q. Zhang Y. . (2024). Motor symptom machine rating system for complete MDS-UPDRS III in Parkinson’s disease: a proof-of-concept pilot study. Chin. Med. J. 137, 1632–1634. doi: 10.1097/CM9.0000000000003044, 38501363 PMC11230756

