# Supplementary material for: Machine learning models for the prediction of levodopa response to tremor in Parkinson’s disease
Source: Front Aging Neurosci. 2026 Jan 5;17:1690155. doi: 10.3389/fnagi.2025.1690155 (PMC12813012; doi:10.3389/fnagi.2025.1690155)
Supplement: Supplementary file 1 [file Table_1.DOCX]

Supplementary Material

This file includes:

Cluster Analysis

eTable 1 Comparisons between two clusters

**Cluster Analysis**

A data-driven clustering approach was developed to divide the training dataset (as described in Section 2.4.1) into two clusters using the following features: ΔLR tremor score, ΔLR postural tremor score, ΔLR action tremor score, ΔLR resting tremor score, and their corresponding percentage changes (%ΔLR). To reduce dimensionality and enhance interpretability, principal component analysis (PCA) was applied to these features. The number of principal components (n_pcs) was varied from 1 to 8 in increments of 1 to determine the optimal number of components to retain.

For each n_pcs setting, four clustering algorithms were applied: Hierarchical Clustering, K-means, Bisecting K-means (biKMeans), and Partitioning Around Medoids (PAM). The Silhouette Index was used as the clustering evaluation metric to assess how well data points fit within their assigned clusters(Rousseeuw, 1987). For each n_pcs value, the algorithm yielding the highest Silhouette Index was considered the optimal clustering method. The final clustering configuration was selected based on the highest Silhouette Index across all n_pcs settings. Our results showed that K-means clustering with n_pcs = 2 achieved the best performance, with a Silhouette Index of 0.386, and was therefore adopted as the final clustering configuration.

Significant differences in both the ΔLR tremor score and the %ΔLR tremor score were observed between the two resulting clusters (see eTable 1). To identify the optimal cutoff values for these two features, receiver operating characteristic (ROC) curve analysis was conducted using the cluster-derived subgroup labels as the reference. Specifically, the actual ΔLR tremor score (U3_Tremor_change_selected) and the %ΔLR tremor score were each used as predictors in separate analyses to evaluate their ability to discriminate between the two subgroups identified by unsupervised clustering, representing levodopa-responsive and levodopa-resistant tremor profiles. The optimal cutoff value for %ΔLR tremor score was 0.519 (AUC = 0.853), while the optimal cutoff value for ΔLR tremor score was 2.5 (AUC = 0.931). To determine whether the difference in AUCs was statistically significant, DeLong’s test was applied. The results showed that the AUC for the ΔLR tremor score was significantly higher than that for the %ΔLR tremor score (p = 0.0086), indicating superior discriminative performance. As a result, the ΔLR tremor score was selected as the criterion for defining levodopa responsiveness due to its stronger classification accuracy.

To enhance clinical applicability, the cutoff value for the ΔLR tremor score was rounded from 2.5 to 3. Accordingly, levodopa-responsive tremor was defined as a ΔLR tremor score >3, a threshold that balances statistical optimization with practical clinical decision-making. Based on this threshold, patients with a ΔLR tremor score greater than 3 were classified as having levodopa-responsive tremor, whereas those with a score ≤3 were classified as having levodopa-resistant tremor.

**eTable 1 Comparisons between two clusters**

|  | Cluster 1 | Cluster2 | *P*^a^ |
| --- | --- | --- | --- |
| n | 96 | 61 |  |
| %ΔLR tremor score | 0.33 (0.31) | 0.75 (0.23) | <0.001 |
| ΔLR tremor score | 1.99 (2.14) | 7.46 (3.44) | <0.001 |
| %ΔLR postural tremor score | 0.09 (0.23) | 0.64 (0.42) | <0.001 |
| ΔLR postural tremor score | 0.16 (0.39) | 1.43 (1.12) | <0.001 |
| %ΔLR action tremor score | 0.11 (0.29) | 0.53 (0.43) | <0.001 |
| ΔLR action tremor score | 0.17 (0.43) | 1.00 (0.88) | <0.001 |
| %ΔLR resting tremor score | 0.33 (0.39) | 0.72 (0.32) | <0.001 |
| ΔLR resting tremor score | 1.67 (2.18) | 5.03 (3.77) | <0.001 |

Note: Data are shown as mead (sd)

^a^ *P* was estimated using Shapiro‒Wilk test. *P* less than 0.05 were indicated significant differences between two clusters.

**Reference**

Rousseeuw, P.J. (1987). Silhouettes: A graphical aid to the interpretation and validation of cluster analysis. *Journal of Computational and Applied Mathematics* 20**,** 53–65. doi: <https://doi.org/10.1016/0377-0427(87)90125-7>.
